# A case of posterior mediastinal myelolipoma and a literature review of its imaging manifestations

**DOI:** 10.1186/s13019-024-02829-1

**Published:** 2024-07-18

**Authors:** Long Xu, Xing Wen, Shi Yan Feng

**Affiliations:** 1 Department of Radiology, Ziyang People’s Hospital, No. 576, Checheng Avenue, Ziyang, 641300 Sichuan People’s Republic of China; 2 Department of Gastrointestinal Surgery, Ziyang People’s Hospital, No. 576, Checheng Avenue, Ziyang, 641300 Sichuan People’s Republic of China

**Keywords:** Myelolipoma, Mediastinal, CT

## Abstract

Mediastinal myelolipoma is a rare condition and has no obvious symptoms. In the past 20 years, some clinical cases have been documented. However, the literature has not systematically summarized its imaging features. The aim of this paper is to present a case of right posterior mediastinal myelolipoma and to review and summarize its imaging features. Twenty-six articles were included in our study, which included a total of 26 patients and 33 lesions; 90.9% of the lesions were located in the mediastinum at the level from the 8th thoracic vertebral body to the thoracic 12th vertebral body. Among the cases with unilateral mediastinum, 68.4% of the cases were located in the right posterior mediastinum. Bilateral lesions accounted for almost one-fourth of all lesions. After contrast medium was injected, 93.9% of the lesions had mild to moderate enhancement; 84.8% of the lesions contained fat density; and 75.8%, 69.7%, 87.9%, and 75.8% of the lesions showed clear boundary, regular shape, heterogeneity and were encapsulated, respectively. Only 12.1% of the lesions contained calcification. An inhomogeneous mass in the right posterior mediastinum near the spine, including fat density, is the predominant imaging marker of most mediastinal myelolipomas.

## Introduction

Although mediastinal myelolipoma is rare, it can occasionally be encountered in clinical work. Myelolipoma is a benign tumor composed of mature adipocytes and hematopoietic tissue, that most often occurs in the adrenal gland but can also occur outside the adrenal gland [1]. The incidence of extra-adrenal myelolipoma has been reported to be very low, with an incidence of 0.08%~0.2% [2]. The uncommon primary sites of myelolipoma include the presacral area, retroperitoneum, liver, spleen, stomach, omentum, pia mater, and mediastinum [3]. With the wide application of imaging examinations, the incidence rate of the disease has increased year by year. To date, most of the reports on mediastinal myelolipoma have been case reports, and there have been no reports in the literature that have summarized its imaging features. Our goal here is to present a case of right posterior mediastinal myelolipoma and to review and summarize its imaging findings.

## Case report

The case is a 55-year-old female Han Chinese patient. Three years ago, the patient had no obvious cause; recurrent chest tightness and chest pain, aggravated after activity and relieved after rest; and felt slightly tired, accompanied by dizziness, headache, fatigue, palpitation, epigastric discomfort, acid reflux, and hiccups. Our laboratory results reported: no white blood cell elevation; glycosylated hemoglobin A1c,7.3%(4.1–6.1%); Type B natriuretic peptide precursor, 363.6 pg/ml(0-125pg/ml); creatinine,122.4 umol/l(31–97 umol/l); urea nitrogen, 9.52 mmol/L(2.9-8.2mmol/L); uric acid,551.0 umol/l(140–380 umol/l); endogenous creatinine clearance rate, 30.56 ml/min(70-200 ml/min); triglyceride, 3.14 mmol/l(0.2–1.7 mmol); blood glucose, 16.67 mmol/l(3.9-6.1mmol/l); neutrophil percentage, 77.00%(40–75%); whole course C-reactive protein, 8.42 mg/L(0–4.0 mg/L). There were no abnormalities found during a physical examination. The previous treatment was effective according to “coronary heart disease”, but the condition had returned. The patient had hypertension, type 2 diabetes, and hyperlipidemia but no anemia.

**Fig. 1:**
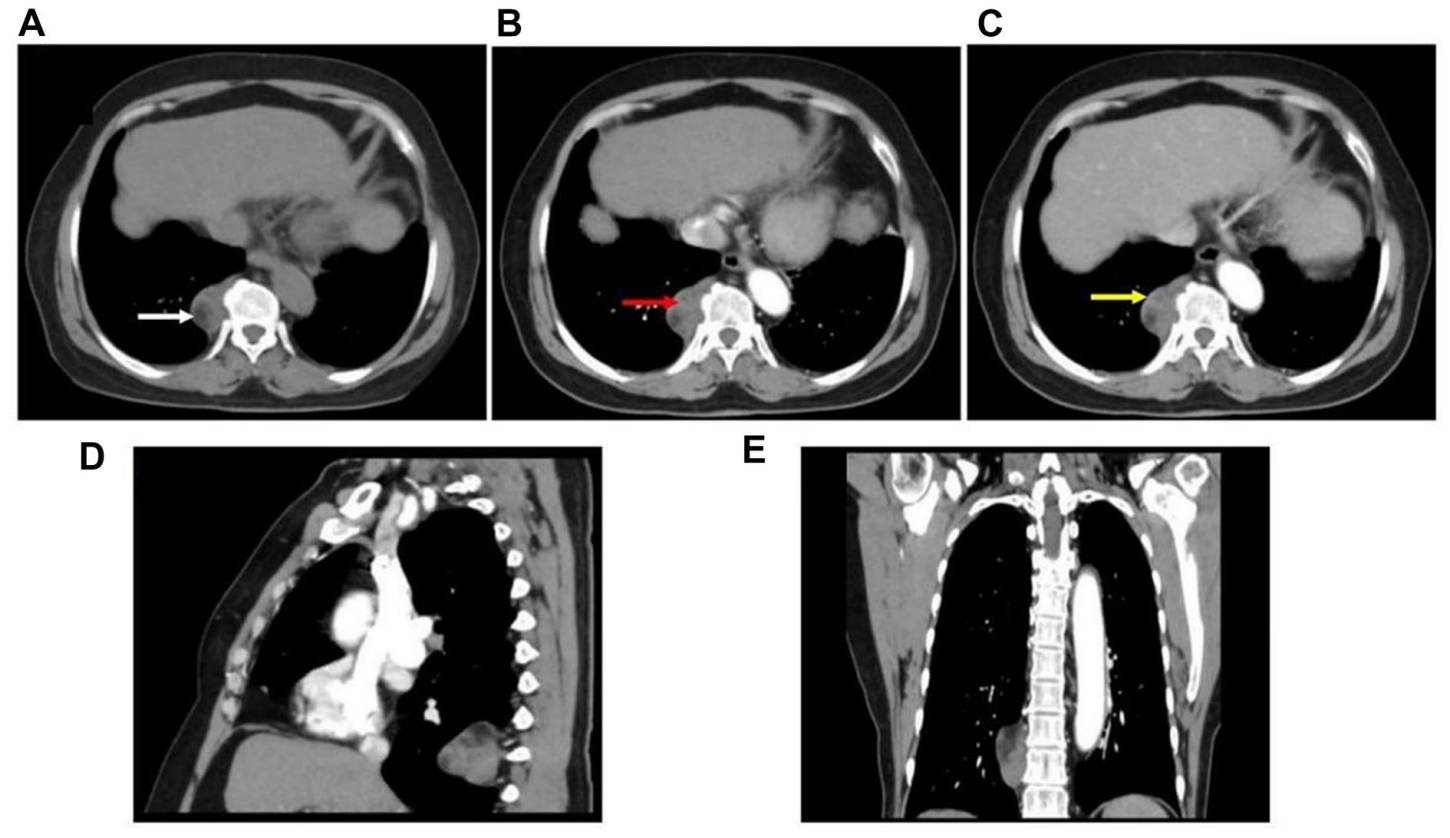
CT image of a right posterior mediastinal myelolipoma: The white arrow in (**A**) shows the fat composition; the red arrow in (**B**) shows mild enhancement (the common CT value in this area is 45Hu, 58Hu in arterial phase, and 67Hu in venous phase). The yellow arrow in (**C**) shows the capsule

A CT image showed an irregular mass of mixed density on the right side of the 9th-10th thoracic vertebral bodies. The mass had a regular shape, clear boundaries and a capsule, some of which were fat density; and a size of approximately 2.8 × 2.5 cm, slightly enhanced after enhancement (Fig. 1). No pulmonary disease was found, no mediastinal lymph node enlargement was found, and no pleural effusion was found. The doctor first diagnosed schwannoma before surgery.

**Fig. 2:**
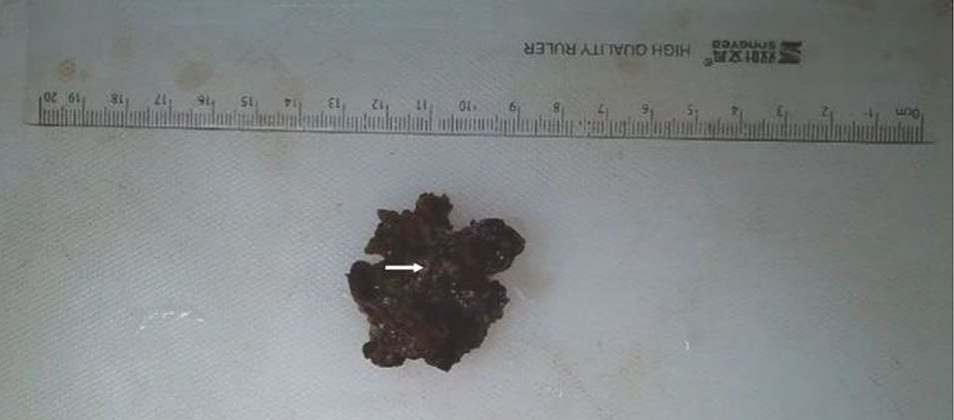
The image shows the surgically removed myelolipoma. White arrows show pale yellow adipose tissue

**Fig. 3:**
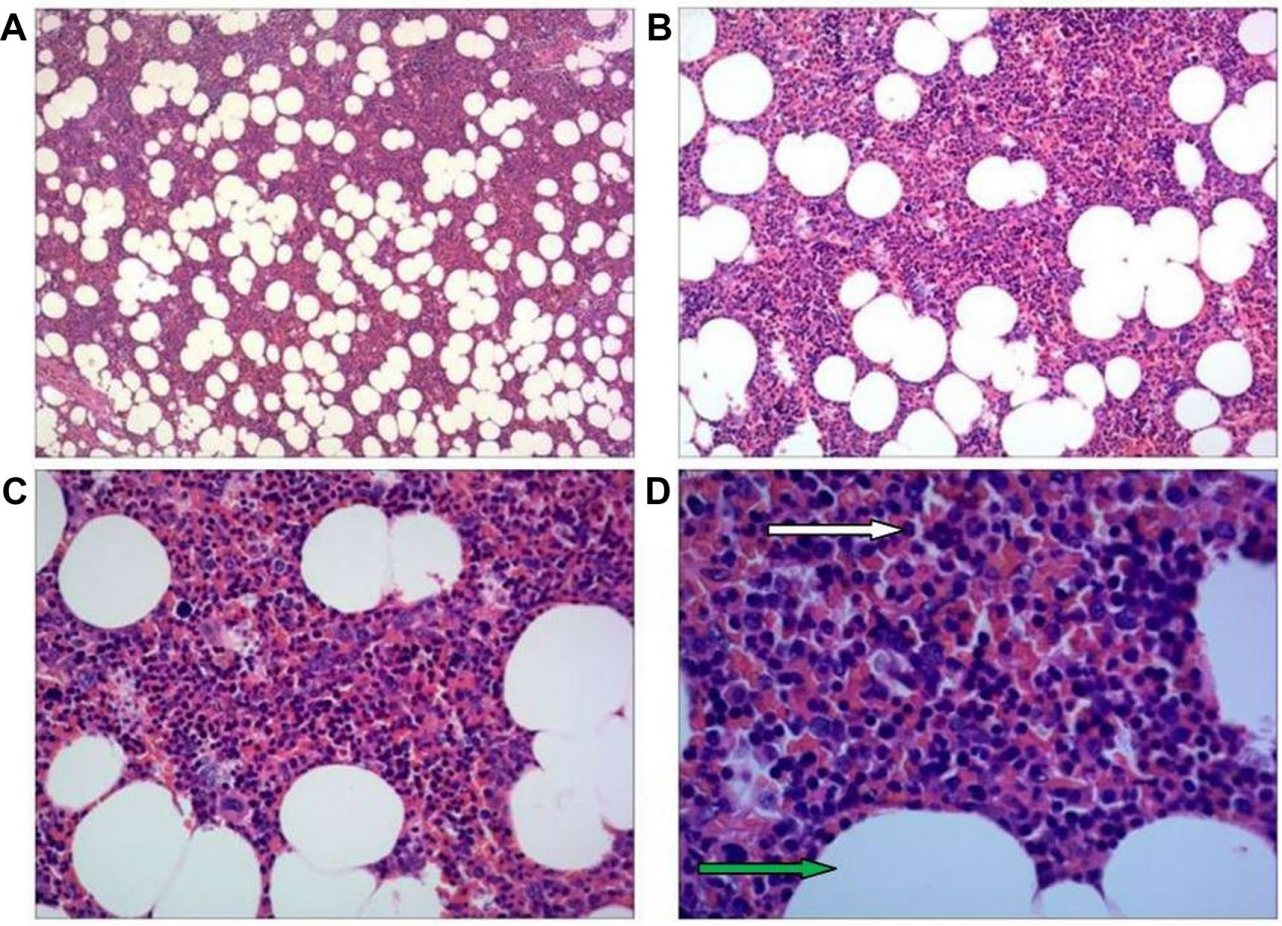
(**A**–**D**) Magnifications of 40x, 100x, 200x, and 400x of myelolipoma under the microscope, respectively(H&E). Microscopic examination showed that the tumor was composed of mature adipose tissue mixed with mature hematopoietic tissue. The white arrow indicates mature hematopoietic tissue. The green arrow indicates adipocytes

The video-assisted thoracic surgery (VATS) intraoperative findings showed a 2.5 cm x 2.0 cm tumor in the right posterior lower mediastinum near the 9th-10th thoracic vertebral bodies, with a clear boundary and complete capsule. It was adjacent to the thoracic sympathetic nerve chain, and the blood vessels on the surface of the lesion were dilated. Fish-like organisms were observed after cutting the capsule, with obvious bleeding. The pathological results showed myelolipoma (Figs. 2 and 3).

**Fig. 4:**
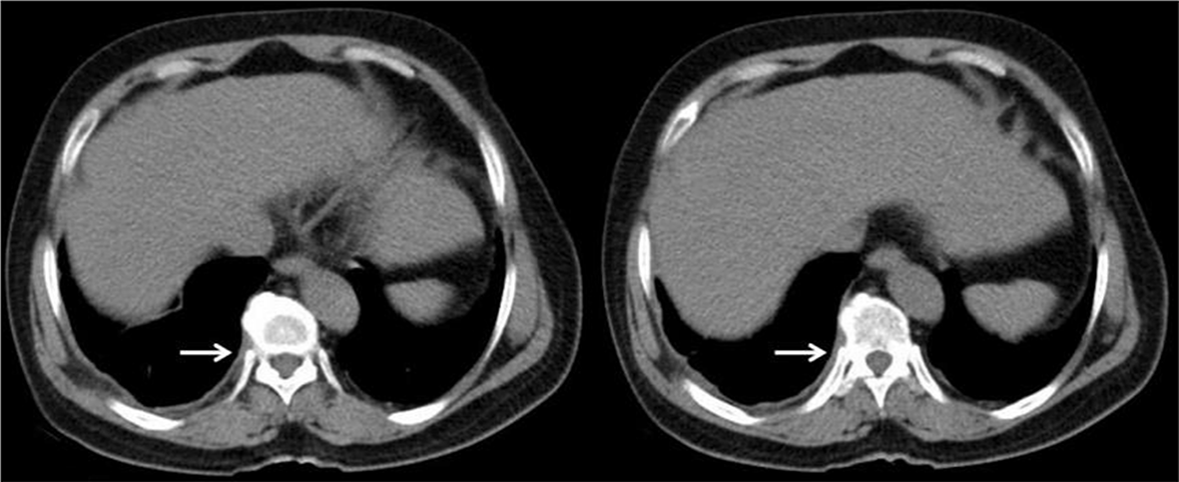
This is a two-slice chest CT image of the patient 4 months after surgery. The white arrow shows that the previous mass is no longer present, no tumor recurrence is seen, and there is pleural thickening after surgery

After the operation, we rechecked the patient’s chest CT image. We reviewed the patient’s chest CT images one month and four months after surgery. The patient recovered well after the operation, and no tumor recurrence was found in the area of the operation. The following figure shows the chest CT image of the patient, 4 months after surgery (Fig. 4).

## Literature review

We searched the literature in PubMed, Web of Science, and the Cochrane Library databases using the subject words myelolipoma and mediastinal. From April 1984 to September 2023, 39 relevant articles were searched, and 26 articles were included according to the integrity of article information and data. For the included cases, the basic information was required to be complete, and the images of the cases had to be described in detail or detailed information could be obtained from the images. For example, cases where the location of the lesion could not be accurately obtained were excluded.

A total of 26 patients and 33 lesions were included in the study. The age ranged from 33 to 79 years (60.8 ± 12.2). There were 6(23.1%) females and 20(76.9) males. The sizes of the lesions were approximately 20–93 mm (44.4 ± 20.5); 30(90.9%) lesions were located at the level of the 8th–12th thoracic vertebral bodies. For cases with unilateral mediastinum, the lesions in 13(68.4%) cases were located in the right posterior mediastinum; the lesions in two(10.5%) cases were located in the left posterior mediastinum; the lesions in two(10.5%) cases were located in the right middle mediastinum; and the lesions in two(10.5%) cases were located in the left and right anterior mediastinum. Lesions were found in the bilateral posterior mediastinum in seven(26.9%) cases;31(93.9%) lesions showed mild to moderate enhancement; 28 (84.8%) lesions contained lipid density ; calcification was found in four(12.1%) lesions; 25 (75.8%) lesions had clear boundaries; the morphology of 23(69.7%) lesions was regular; 29 (87.9%) lesions were heterogeneous; and 25(75.8%) lesions were encapsulated (Table 1).

**Table 1:** Imaging features of mediastinal myelolipoma in 26 patients

Characteristics	Number	Percentage
Unilateral lesions/bilateral lesions/total lesions	19/7/33	
Male	20	76.9%
Level of thoracic 8 vertebral body to thoracic 12 vertebral body	30	90.9%
Right posterior mediastinum	13	68.4%*
Left posterior mediastinum	2	10.5%*
Right middle mediastinum	2	10.5%*
Left anterior mediastinum	1	5.3%*
Right anterior mediastinum	1	5.3%*
Bilateral posterior mediastinum	7	26.9%
Mild to moderate enhancement	31	93.9%
Lipid density	28	84.8%
Calcification	4	12.1%
Clear boundary	25	75.8%
Regular shape	23	69.7%
Heterogeneous	29	87.9%
Capsule	25	75.8%

* Represents the percentage of cases of unilateral mediastinal lipoma, excluding cases of bilateral mediastinal lipoma

## Discussion

Most myelolipomas have no clinical symptoms. Most of them are accidentally found during physical examination or imaging examination due to other diseases [4]. In addition, in most patients, mediastinal myelolipoma appears around the age of 60, and there has been no significant relationship reported between gender and tumors [5, 6]. Similarly, in our study, the highest incidence rate was approximately 60 years old. However, we found that the incidence rate in males was significantly higher than that of in females. This was inconsistent with previous studies and may have been related to the fewer cases and statistical errors in previous studies.

Mediastinum accounts for approximately 3% of all myelolipomas and is mainly located in the lower right posterior mediastinum [5]. In our study, 68.4% of the cases were located in the right posterior mediastinum. Mediastinal myelolipoma can be single or multiple and located on one or both sides of the posterior mediastinum [7, 8]. In rare cases, it has been located in the anterior mediastinum; however, the study did not mention the image features [9]. In our study, most cases were unilateral lesions and 26.9% of the cases were bilateral lesions.

According to one study, the CT value of the tumor, which included fat and bone marrow tissue, was 20–50 Hu. In the case of the contrast agent, tumor enhancement was not obvious. In addition, calcification in tumors is rare [10]. This is roughly consistent with our study. In our study, fat-like density could be seen in most lesions (84.8%). After contrast enhancement, most lesions (93.9%) had mild to moderate enhancement, and only a few lesions (12.1%) showed calcification. Most lesions were heterogeneous and encapsulated.

There is little information on MRI-related reports of mediastinal myelolipoma. Because tumors contain fat components, most tumors have high signal intensity on T1WI and T2WI [11]. According to the main content of the mass, MRI may show equal intensity or slightly higher intensity in T1- and T2-weighted images [6]. One researcher mentioned that the MRI signal of mediastinal myelolipoma could be divided into three types according to the tumor components: fat signal, adult cortex signal, and signal between fat and liver [12].

CT is a common diagnostic method for mediastinal myelolipoma. CT and MRI can effectively diagnose adrenal myelolipoma, but they cannot diagnose mediastinal myelolipoma [13]. Even if the disease cannot be accurately diagnosed, it can be judged whether the disease is benign or malignant by using imaging [11]. Different tumors can be distinguished by their occurrence in distinct locations and the different tissue components present within a tumor. In particular, mediastinal myelolipoma needs to be differentiated from teratoma, neurofibroma, schwannoma, malignant nonseminomatous germ cell tumors, thymolipoma, angiolipoma, and epidural lipomatosis [14].

Adrenal myelolipoma is relatively common. It often occurs unilaterally, with a small volume, clear boundary, circular homogeneous mass, and often contains fat [15, 16]. It is notable that the amount of fat is much less in mediastinal myelipoma than in adrenal myelipoma.

When a paraspinal posterior mediastinal mass is found, especially if the mass is located in the right posterior mediastinum, in addition to the clear boundary and regular shape of the benign tumor signs, if the mass contains fat density, the possibility of myelolipoma should be considered first. Except for special situation such as extremely large masses, internal bleeding, and compression symptoms, radiologists should recommend follow-up rather than biopsy or surgery [17, 18].

General laboratory tests for myelolipoma show no abnormalities, and CT and MRI are the most important preoperative diagnostic methods; CT is more commonly used than MRI. In this study, some radiographic signs were characteristic, with the main radiographic sign being a heterogeneous mass in the right posterior mediastinum near the spine, including fat density. When the imaging features of the lesions are not typical, they mainly need to be differentiated from neurogenic tumors, extramedullary hematopoiesis, lymphadenopathy, and pleural mesothelioma.

## Data Availability

These data and materials are available from corresponding authors for rational reasons.
